# Biophysical insights into plasmid DNA binding by d*einococcus grandis* Dps nanocage

**DOI:** 10.1007/s00249-026-01830-x

**Published:** 2026-03-06

**Authors:** Ana J. Carvalho, M. Raquel Pacheco, João P. L. Guerra, Nykola C. Jones, Søren V. Hoffmann, Tomás Calmeiro, Elvira Fortunato, Alice S. Pereira, Pedro Tavares

**Affiliations:** 1 UCIBIO − Applied Molecular Biosciences Unit, Department of Chemistry, NOVA School of Science and Technology, Universidade NOVA de Lisboa, Caparica, 2829-516 Portugal; 2 Associate Laboratory i4HB - Institute for Health and Bioeconomy, NOVA School of Science and Technology, Universidade NOVA de Lisboa, Caparica, 2829-516 Portugal; 3 ISA, Department of Physics and Astronomy, Aarhus University, Aarhus C, DK-8000 Denmark; 4 CENIMAT/i3N, Department of Materials Science, NOVA School of Science and Technology, Universidade NOVA de Lisboa, Caparica, 2829-516 Portugal; 5 Department of Molecular Biology and Genetics, Aarhus UniversityUniversitetsbyen, Aarhus C, 81, 8000 Denmark

**Keywords:** DNA-binding protein from starved cells (Dps), Protein nanocages, Protein-DNA interaction, Synchrotron radiation circular dichroism (SRCD) spectroscopy

## Abstract

DNA-binding proteins from starved cells (Dps) are small multifunctional protein nanocages expressed by prokaryotes under oxidative stress or during starvation, acting as a key bacterial defense mechanism. Dps proteins protect DNA either through direct binding or by scavenging reactive oxygen species precursors. In most Dps homologs studied to date, DNA-binding is mediated by flexible, intrinsically disordered N- or C-terminal extensions. In this study, we investigated the interaction of *Deinococcus grandis* Dps (DgrDps) and a mutant variant, lacking the first 46 N-terminal residues, with supercoiled plasmid pUC19 using electrophoretic mobility shift assays (EMSA), DNase I protection assays, atomic force microscopy (AFM), and synchrotron radiation circular dichroism (SRCD). DgrDps binds supercoiled pUC19 with an apparent dissociation constant (*K*_D_) of 5.2 ± 0.3 µM, exhibiting positive cooperativity. Our results indicate that DNA binding is primarily mediated by the flexible N-terminal tails of DgrDps. AFM imaging revealed that DgrDps binds to multiple sites on the plasmid, inducing DNA bridging and compaction. Furthermore, the presence of 96 Fe^2+^/dodecamer increased the compaction of these protein-pUC19 complexes. This organization confers physical protection against DNase I digestion. Additionally, SRCD spectroscopy provided insights into the structural features and thermal stability of the DgrDps-DNA complexes.

## Introduction

The genus *Deinococcus* comprises non-spore-forming bacteria known for their remarkable resistance to diverse environmental stresses, particularly radiation and desiccation, facilitated by highly efficient DNA repair mechanisms, and their ability to thrive over a wide temperature range (1 to 55 °C) (Peter Hirsch et al. 2004; Cox and Battista 2005). Since their discovery in 1956, these extraordinarily radiation-resistant organisms have been the subject of extensive biological research aimed at elucidating the mechanisms underlying their resilience. These investigations have revealed not only their highly efficient DNA repair pathways, but also protective protein and metabolite networks, antioxidant defense systems, and unique cellular strategies that safeguard genome integrity (Lee et al. 2021; Liu et al. 2023). In response to hazardous stimuli, such as irradiation, these bacteria extensively modulate their gene expression, as revealed by transcriptome analyses of *Deinococcus radiodurans*. This includes the induction of hundreds of genes and the repression of many others during recovery from ionizing radiation. Among the differentially regulated genes, DNA-binding proteins from starved cells (Dps), known for their ability to bind DNA and mitigate oxidative damage, emerge as important contributors to the cell’s defense mechanisms (Wang et al. 2019; Chen et al. 2022; Bae et al. 2023). While some studies have shown that Dps is upregulated upon ionizing radiation, thus implying a role in genome protection during stress, its exact regulatory dynamics and comprehensive contribution remain the subject of ongoing research.

**Fig. 1:**
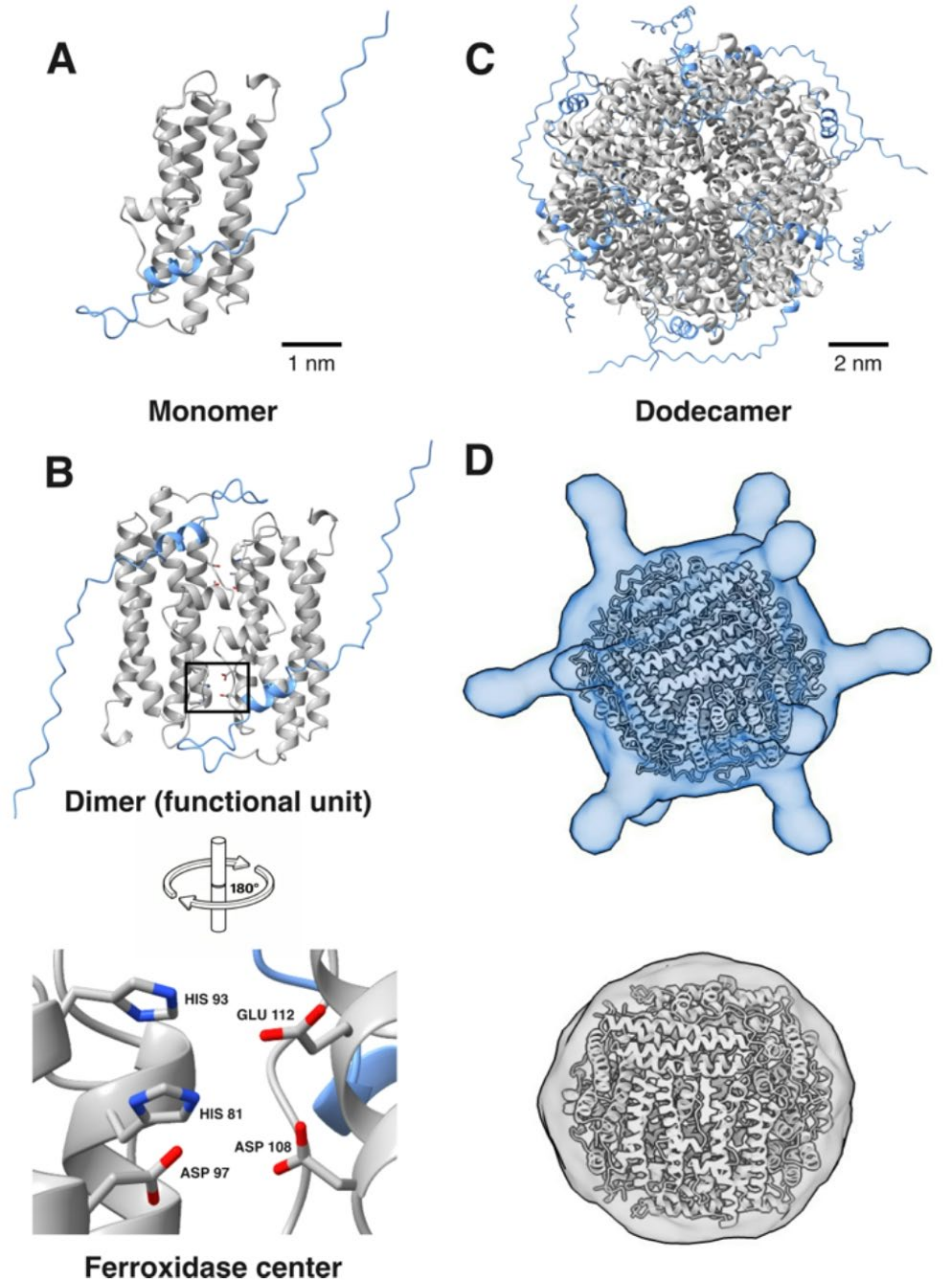
– Structure of *Deinococcus grandis* Dps (DgrDps). (A) Model of the DgrDps monomer, with its four-helix bundle motif in gray and the N-terminal tail in blue. (B) DgrDps dimer, highlighting the two ferroxidase centers (enlarged below, showing the conserved FOC residues as sticks). (C) Quaternary structure of DgrDps, a spherical, cube-shaped dodecamer formed by self-assembly of the monomers. These models were obtained via homology modeling (SwissModel), using *D. radiodurans* Dps1 (PDB 2C2F) as template. (D) Representative ab initio models of both DgrDps wild-type (WT) protein with the elongated N-terminal regions (generated by GASBOR) and its ΔN variant (generated by DAMMIN). The models were derived from SAXS data collected in 50 mM MOPS, pH 7.0 and 230 mM NaCl, and are superimposed with the dodecamer model (ribbons). The SAXS envelope of DgrDps WT shows the N-terminal tails protruding from the dodecamer (Guerra et al. 2023)

DNA protection by Dps proteins is achieved through two interconnected mechanisms: direct DNA protection via binding and condensation, and indirect prevention of oxidative damage through ferroxidation and iron mineralization, reducing the formation of reactive oxygen species via Fenton chemistry. Expression of the *dps* gene is predominantly stimulated during the starvation-induced stationary phase, when Dps becomes the most abundant nucleoid-associated protein. Its first crystallographic structure revealed that Dps exhibits the characteristic four-helix bundle fold of the Ferritin family, as depicted in Fig. 1 (Grant et al. 1998; Guerra et al. 2022). To date, more than a thousand Dps-like proteins have been identified, with approximately 97% found in Bacteria and the remaining 3% in Archaea (Haikarainen and Papageorgiou 2010). While not all members of the Dps family bind DNA, they all possess ferroxidase centers (located at every dimer interface) that allow them to oxidize and mineralize iron.

The direct interaction with DNA occurs through long, flexible, and positively charged N- or C-terminal extensions that facilitate non-sequence-specific electrostatic binding to the negatively charged DNA backbone (Guerra et al. 2021).

**Fig. 2:**
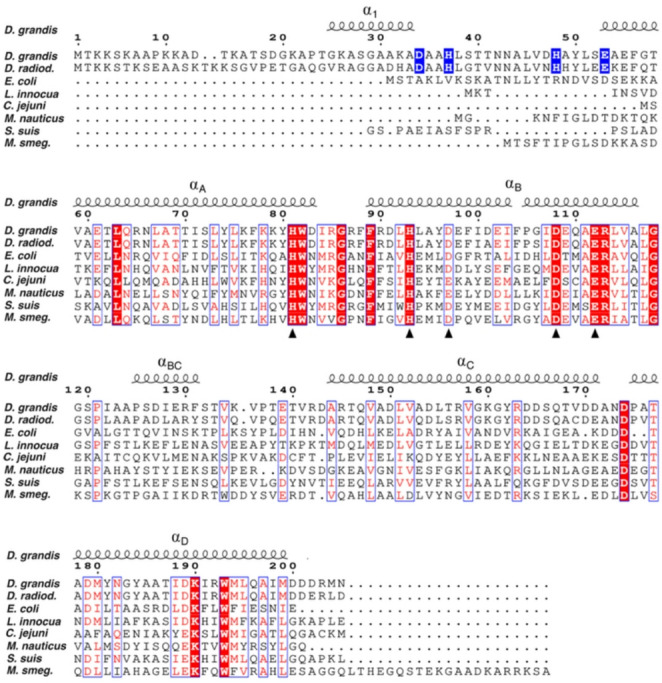
– Comparative sequence alignment of Dps proteins. Alignment of *Deinococcus grandis* (*D. grandis*) Dps with other known Dps proteins from various bacterial species, including *Deinococcus radiodurans* (*D. radiod.*), *Escherichia coli* (*E. coli*), *Listeria innocua* (*L. innocua*), *Campylobacter jejuni* (*C. jejuni*), *Marinobacter nauticus* (*M. nauticus*), *Streptococcus suis* (*S. suis*), and *Mycobacterium smegmatis* (*M. smeg*.). The alignment was prepared using Clustal Omega and ESPript – https://endscript.ibcp.fr (Robert and Gouet 2014; Madeira et al. 2024). Secondary structure is shown above the sequence alignment, where ⍺_A_, ⍺_B,_ ⍺_C_ and ⍺_D_ represent the typical four-helix bundle with a smaller helix midway, characteristic of Dps proteins (Guerra et al. 2021), and an extra small helix (⍺_1_) only present in the N-terminal tail of Dps from *Deinococcus* genus. Strict sequence identity is indicated by a red box with white characters, whereas conserved residues are depicted in red characters. Blue boxes with white characters denote the residues of the N-terminal metal-binding site discovered in *Deinococcus* genus Dps(Guerra et al. 2023) and the conserved amino acids that compose the ferroxidase centers are highlighted by triangles

A sequence comparison of selected Dps proteins is shown in Fig. 2. This comparison highlights finding such as those from *Mycobacterium (M.) smegmatis* Dps, where deletion of the last 16 residues resulted in the loss of DNA binding activity without disrupting the dodecamer structure (Roy et al. 2007). Additionally, protonation of a histidine tag engineered at the C-terminus of the *M. smegmatis* Dps induced DNA condensation through protein self-aggregation, in contrast to the wild-type untagged protein, which only formed smaller complexes, as revealed by atomic force microscopy (AFM) analysis (Ceci et al. 2005).

Ceci and co-workers proposed that Dps self-aggregation and DNA condensation are processes directly correlated with the nature of the N-terminal extensions. More specifically, the number of lysine residues in these regions appears to determine whether the interaction with DNA will lead to the formation of large complexes. Furthermore, the positive charge of the N-terminal extensions not only dictates self-aggregation, but also the mode of interaction with DNA. The *Escherichia coli* Dps demonstrated the capacity for self-aggregation and DNA condensation within the pH range of 6.3 to 8.2. At physiological pH, a mutant lacking the first 8 N-terminal residues (including two lysines) could still bind DNA without condensation. However, removal of the 18 N-terminal residues (comprising three lysine residues) abolished DNA binding (Ceci et al. 2004). In *Lactococcus lactis*, the helical N-terminal tails of DpsA and DpsB were found to directly mediate DNA binding (Stillman et al. 2005). In contrast to *L. lactis* Dps, the N-terminal tails of *D. radiodurans* Dps1 are predominantly disordered, a feature common to the tails of almost all Dps described so far. Guerra and collaborators demonstrated that the flexible N-terminal tails of DgrDps can adopt a range of dynamic conformations modulated by ionic strength (Guerra et al. 2022). The present study elucidates the DNA-binding properties of the unusually long N-terminal tails of DgrDps. To understand their role, a tailless variant (termed ΔN) lacking the first 46 amino acid residues, served as a comparative tool. DNA binding to supercoiled plasmid pUC19 was assessed using electrophoretic mobility shift assays (EMSA), atomic force microscopy (AFM), and synchrotron radiation circular dichroism (SRCD). The impact of Fe^2+^ ions, the protein’s substrate, on DNA binding was evaluated. Protection assays utilizing DNase I and Proteinase K were also performed. Lastly, the thermal stability of the protein-DNA complexes was examined by SRCD spectroscopy.

## Results and discussion

### Electrophoretic mobility shift assays

**Fig. 3:**
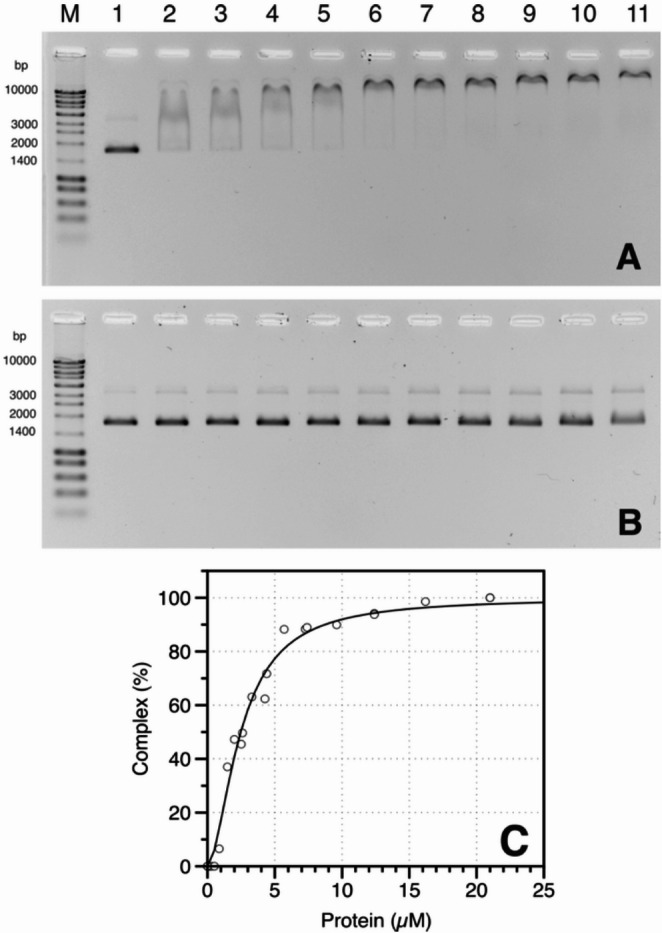
– EMSA of DgrDps proteins binding to supercoiled pUC19 DNA, in 50 mM MOPS, pH 7.0 and 230 mM NaCl buffer. Agarose gels of DgrDps WT (A) and ΔN variant (B). M – NZYDNA Ladder III; 1 – pUC19 control (7 nM); 2 to 11 – Binding reactions containing pUC19 (7 nM) incubated with increasing concentrations of DgrDps proteins, ranging from 0.2 to 21 µM. C) Hill plot of DgrDps WT binding to pUC19. Fractional complex formation, derived from migration distance, is presented for two independent data sets. The solid line on top of the experimental data points represents a theoretical fit using the Hill equation

The ability of DgrDps proteins to bind pUC19 was initially evaluated using agarose gel electrophoresis (Fig. 3). In the absence of protein, supercoiled plasmid pUC19 (2686 bp) exhibited a single predominant band upon electrophoresis (Lane 1 in Fig. 3, A and B). The addition of DgrDps resulted in the formation of large protein-DNA complexes that migrated slower than the free supercoiled pUC19 (Fig. 3A, lanes 2 − 12). Maximum complex formation was achieved at DgrDps concentrations above 5.7 µM. DgrDps ∆N, the tailless protein variant, did not form detectable complexes with pUC19, as shown by the lack of an electrophoretic mobility shift compared to the free plasmid DNA band (Fig. 3B). Maximum condensation of pUC19 with *Deinococcus radiodurans* Dps-1 was achieved at a 500-fold protein excess (4.5 µM DrDps1, 9 nM pUC19) in 40 mM Bis-Tris pH 6.5 with 150 mM NaCl after 15 min of reaction, compared to the 800-fold excess observed in this work at pH 7.0 (Santos et al. 2015). Lowering the pH would increase the protonation of specific groups on the N-terminal extensions of the protein, leading to stronger electrostatic interactions and a greater binding affinity of Dps to DNA. The fractional complex formation was plotted against protein concentration and fit with the Hill equation (Fig. 3C). This analysis yielded an apparent dissociation constant, *K*_D_, of 5.2 ± 0.3 µM and a Hill coefficient, *n*, of 1.8 ± 0.1. These results suggest that DgrDps binds to DNA, leading to a gradual retardation of the pUC19 population until saturation. The Hill coefficient (*n* > 1) confirms positive cooperativity in DNA binding. This finding is comparable to the *n* value of 1.2 ± 0.1 reported for *Marinobacter nauticus* Dps (MnDps, formerly *Marinobacter hydrocarbonoclasticus*; Tindall 2020), which binds pUC19 with a *K*_D_ = 6.0 ± 1 µM (Jacinto et al. 2021). However, unlike MnDps, which induced plasmid relaxation, DgrDps formed protein-DNA complexes with electrophoretic mobility significantly above the 10,000 bp marker band (Fig. 3A). This observation aligns with the known DNA condensation capacity of DrDps1 which exhibits a long N-terminal tail like DgrDps(Reon et al. 2012) whilst MnDps exhibits a shorter one (~ 10 residues long).

**Fig. 4:**
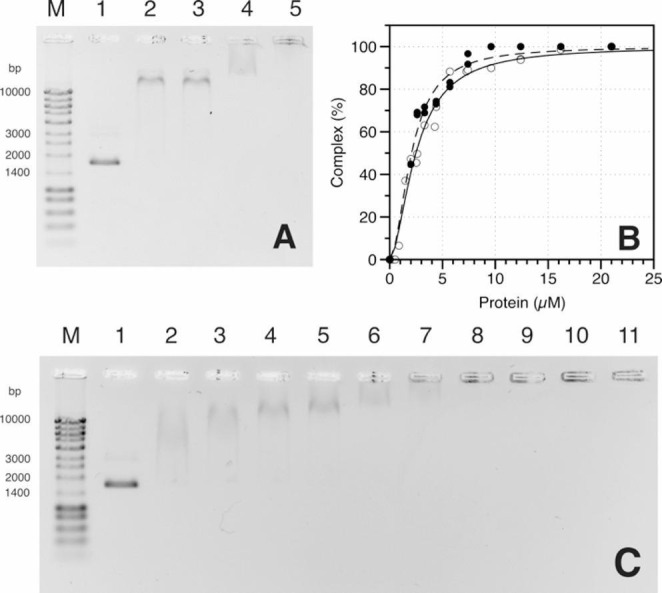
– Impact of iron loading on DgrDps-pUC19 DNA binding analyzed by EMSA. (A) EMSA showing the binding of DgrDps WT, pre-loaded with varying molar ratios of iron/protein (12, 48 and 96 Fe^2+^/DgrDps) under aerobic conditions, to supercoiled pUC19 DNA. 1 – Supercoiled pUC19 DNA; 2 – Apo-DgrDps WT (iron-free) reacted with pUC19; 3 to 5 – DgrDps pre-loaded with 12, 48 and 96 Fe^2+^/protein, respectively, reacted with pUC19. (B) Hill plot analysis comparing the binding of apo-DgrDps WT (empty circles) and DgrDps pre-loaded with 96 Fe^2+^ ions (filled circles) to supercoiled pUC19 DNA. The theoretical fits using the Hill equation are shown as a solid line for the apo-protein and dashed line for the 96 Fe^2+^ loaded protein. (C) Titration of supercoiled pUC19 (7 nM) with DgrDps pre-loaded with 96 Fe^2+^ ions, with protein concentrations ranging from 2.0 to 21 µM. M – NZYDNA Ladder III. Unless otherwise specified, the concentration of DgrDps was 21 µM and pUC19 was 7 nM in all assays

The influence of iron loading on the binding of DgrDps WT to pUC19 was investigated by EMSA using samples containing increasing iron concentrations. As shown in Fig. 4A, the sample loaded with 12 Fe^2+^/Dps exhibited an electrophoretic mobility identical to that of the iron free protein-DNA complex (Fig. 4A, lanes 2 and 3). In contrast, pre-loading with 48 or 96 Fe^2+^/Dps resulted in the formation of large protein-DNA complexes, that were retained within the gel wells, unable to penetrate the gel matrix (Fig. 4A, lanes 4 and 5). Based on these findings, an EMSA was performed with increasing concentrations of the pre-loaded 96 Fe^2+^/Dps (Fig. 4C). Protein-DNA complexes were retained in the wells at a protein concentration above 7.4 µM. The Hill plot (Fig. 4B) revealed *n* = 1.9 ± 0.1 and *K*_D_ = 4.0 ± 0.6 µM, values similar to those of the apo-protein. The key difference is that the 96 Fe^2+^-loaded protein formed aggregates that were excluded from the gel matrix, suggesting that the presence of a ferric iron mineral core contributes to the formation of larger, more condensed protein-DNA complexes. It has been reported that in *Campylobacter jejuni* Dps reaction with Fe^2+^ ions induced DNA binding, believed to be enhanced by Dps self-aggregation (Huergo et al. 2013). Conversely, iron was found to inhibit pUC19 binding by MnDps (Jacinto et al. 2021).

### DNase and protease protection assays

**Fig. 5:**
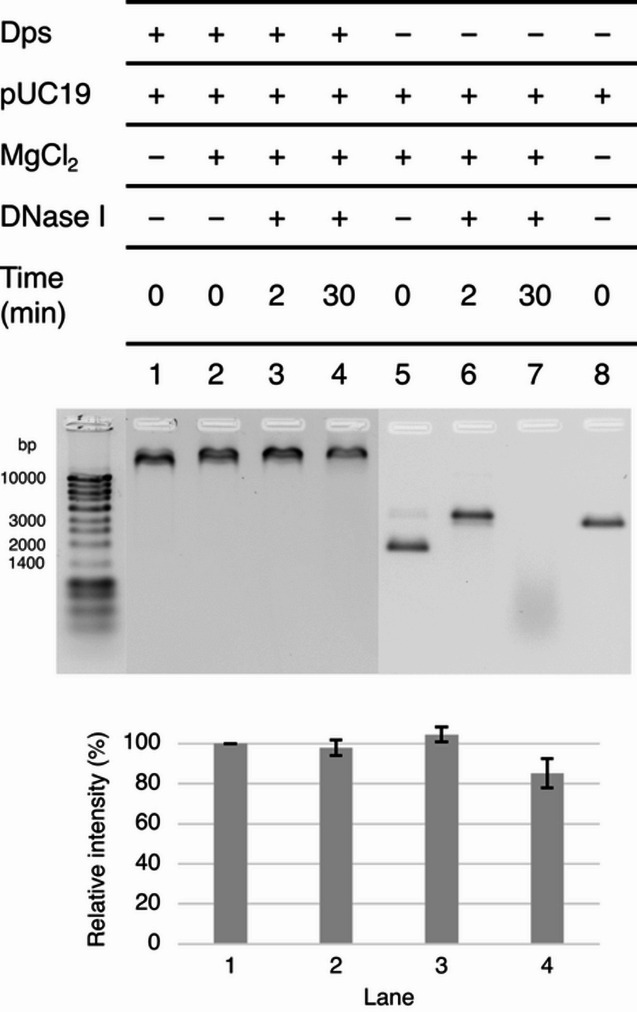
– Protective effect of DgrDps-pUC19 complex against DNase I digestion. pUC19 plasmid DNA (7 nM), either free or pre-incubated with DgrDps WT (21 µM), in the presence of 5 mM MgCl_2_, was reacted with 0.5 µU of DNase I at room temperature. The digestion was monitored after 2 and 30 min of hydrolysis. The composition of each reaction is described above the gel. Lane 8 corresponds to linearized pUC19 for comparison. A graph with the densiometric analysis of selected samples is also shown

To investigate the protective effect of protein-DNA complex formation on both DNA and protein, DNase I and Proteinase K protection assays were performed. The protective capacity of DgrDps on the DNA was evaluated through controlled digestion of pUC19 with DNase I (Fig. 5). The free supercoiled pUC19 control (lane 5) was rapidly converted to its relaxed form within 2 min (lane 6) of DNase I incubation and was completely hydrolyzed after 30 min (lane 7) of reaction. When pUC19 DNA was pre-incubated with DgrDps and subsequently digested with DNase I, no digestion could be observed after 2 min (lane 3). Even after 30 min, only ∼15% of the plasmid DNA was hydrolyzed (lane 4). These results demonstrate that DgrDps WT binding to pUC19 physically shields the DNA from nuclease digestion, consistent with findings for DrDps1 (Reon et al. 2012). Similar protective effects have been reported for other proteins. *Desulfovibrio vulgaris* bacterioferritin, for example, displayed Dps-like protection of pUC19 against DNase I (Timóteo et al. 2012). In the case of *M. smegmatis* Dps, plasmid DNA was protected from DNase activity only when modified with a C-terminal His-tag, which conferred DNA condensation ability (Ceci et al. 2005).

**Fig. 6:**
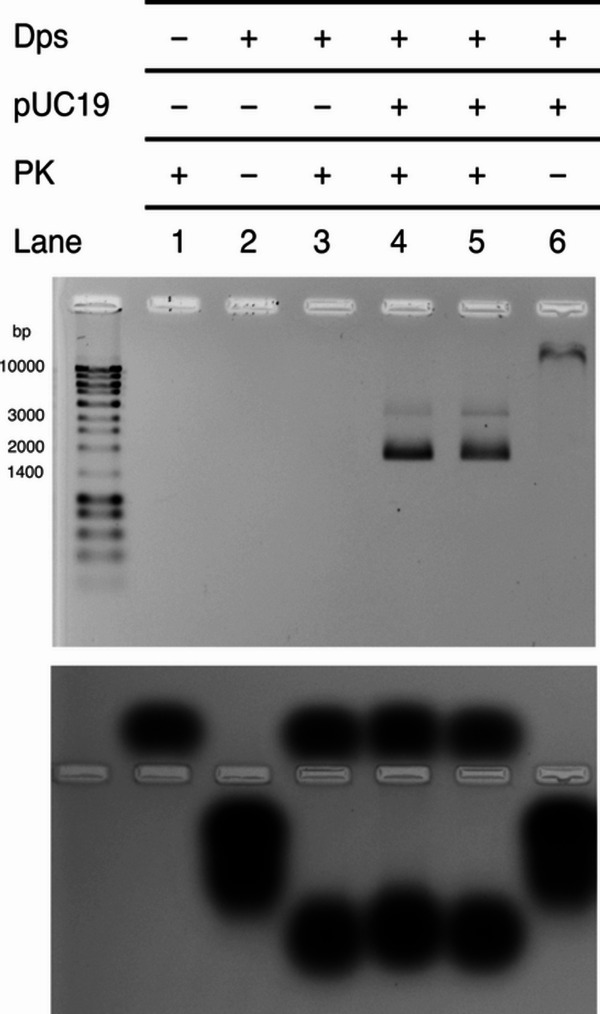
– Protective effect of the DgrDps-pUC19 complex against Proteinase K (PK) hydrolysis. Lane 3 shows a DgrDps (21 µM) proteolytic control without pUC19. In lane 4, pUC19 DNA (7 nM) was added to DgrDps (21 µM) that had been pre-incubated with PK. Conversely, in lane 5, PK was added after DgrDps had bound to pUC19. The bottom panel displays the agarose gel stained with BlueSafe for protein band visualization

To assess if DgrDps protein was protected against proteolysis when bound to pUC19, the protein-DNA complex was digested with Proteinase K, a serine protease with a broad substrate specificity (Fig. 6).

As expected, the free protein was hydrolyzed when incubated with Proteinase K (lane 3), consequently impairing its ability to interact with pUC19 (lane 4). Incubation of the protein-DNA complex with Proteinase K disrupted the complex (lane 5), indicating that the pUC19 plasmid does not shield the protein from proteolysis. Staining the EMSA gel for protein detection showed that the hydrolyzed Dps band had increased mobility compared to the free protein band, suggesting a peptide site susceptible to proteolysis.

## Atomic force microscopy

**Fig. 7:**
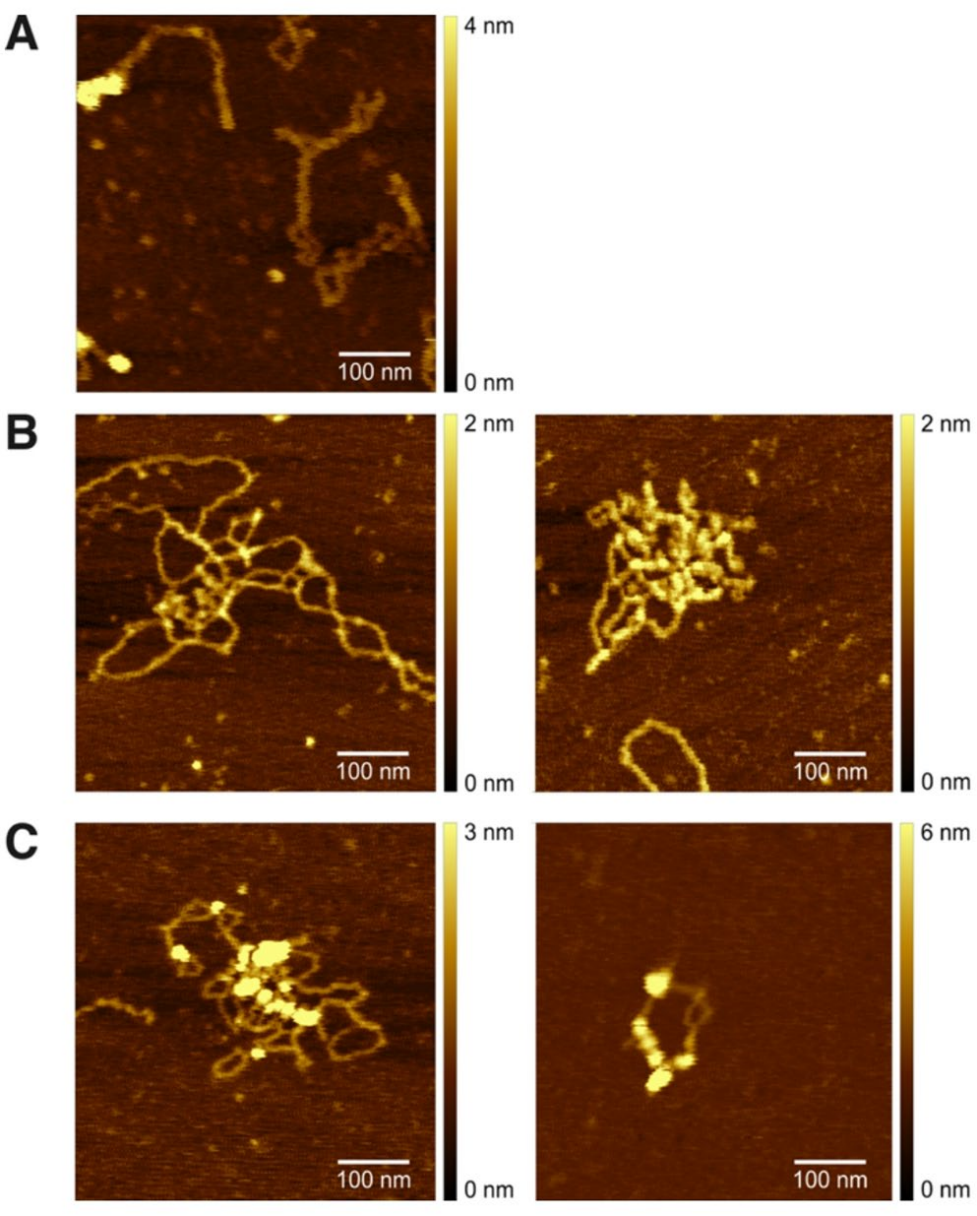
– AFM imaging of DgrDps binding to supercoiled pUC19 DNA. (A) pUC19 control; (B) DgrDps WT incubated with pUC19; and (C) DgrDps containing a ferric iron mineral core (96 Fe^2+^/dodecamer) and reacted with pUC19. The mica surface was pre-incubated with 5 mM NiCl_2_ to facilitate DNA deposition. AFM images in B) and C) are of the same sample acquired at two different locations within the same scan

The topology of the protein–DNA complexes was characterized by atomic force microscopy (AFM) and compared with the free form of plasmid DNA (Fig. 7).

The micrograph in Fig. 7A confirmed that pUC19 predominantly adopted a supercoiled conformation, with distinct strands crossing (or bridging spots) visible on the DNA molecule. As shown in Fig. 7B, DgrDps WT incubation with pUC19 led to the protein binding in various regions of the plasmid DNA molecule, promoting its bridging and packaging. Our observations are consistent with previous AFM results for *Escherichia coli* Dps incubated with pUC9-5 S (Ceci et al. 2004).

As the proposed model predicts, initial Dps binding promotes the subsequent recruitment of additional protein molecules, forming a multipoint interaction network. This network bridges distant regions, either within the same molecule or between different molecules, resulting in substantial DNA condensation (Chiancone and Ceci 2010). AFM images of pUC19 incubated with DgrDps loaded with 96 Fe^2+^ ions per dodecamer (Fig. 7C), revealed clear self-aggregation of the protein. This aggregation led to significantly increased DNA compaction compared to the apo-protein, which correlates with the EMSA results.

## Synchrotron radiation circular dichroism

Synchrotron radiation circular dichroism (SRCD) provides enhanced signal quality and improved sensitivity compared to conventional benchtop spectrophotometers, particularly in the far-UV region, due to its high photon flux. This facilitates the detection of significant structural changes, thereby contributing to the characterization of dynamic conformational transitions. In addition, temperature control and the possibility of using different acquisition geometries further enhance the characterization of biomolecular interactions and stability. The data presented in this work were measured at the AU-CD beam line on the ASTRID2 synchrotron light source at Aarhus University, Denmark. Further details can be found in the supplementary information.

### Thermal stability of DgrDps proteins and pUC19

**Fig. 8:**
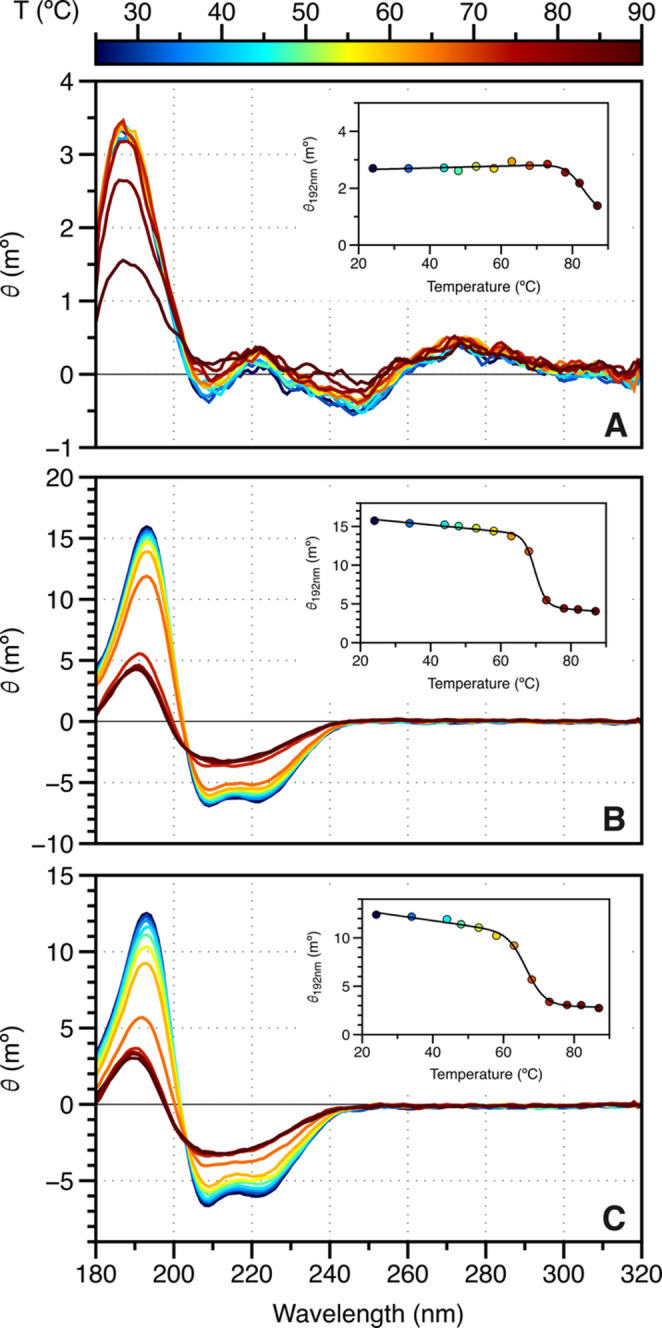
– Thermal stability of pUC19 and DgrDps proteins (WT and ∆N variant) monitored by SRCD. Temperature scans (24 to 87 °C) are shown as stacked spectra for pUC19 (A), DgrDps ΔN (B) and DgrDps WT (C). Thermal denaturation was monitored at 192 nm, with the resulting curves presented as an inset in each panel. The solid lines overlaying the experimental data represent non-linear least-squares fits based on a two-state denaturation model. Samples contained either 0.3 mg/mL of protein (WT or ΔN) or 0.1 mg/mL pUC19, all prepared in 10 mM MOPS (pH 7.0) and 240 mM NaF

The thermal stability of DgrDps proteins (WT and ∆N variant) and pUC19 was assessed by performing SRCD temperature scans from 24 to 87 °C. Figure 8 displays stacked spectra from these scans for each biomolecule, generally showing a shift from a folded spectrum at 24 °C to a mostly unfolded one, with a decrease in the intensity of the overall signal.

At 24 °C, both DgrDps proteins exhibited CD spectra with a maximum at 193 nm and minima at 209 and 222 nm, typical features for proteins with a high α-helical content (Guerra et al. 2021, 2022). The CD spectrum of pUC19 at this temperature exhibited positive bands at 187 nm and around 275 nm and slightly negative bands around 209 and 245 nm. The spectral shape is characteristic of B-DNA, the most common and predominant form of DNA (Grove and Wilkinson 2005).

Thermal denaturation was monitored by plotting the CD signal intensity at 192 nm as a function of temperature (Fig. 8). The thermal denaturation parameters determined by theoretical fitting are presented in Table 1. Plasmid DNA exhibited high temperature tolerance, with structural changes initiating at 75 °C and a melting temperature (T_m_) of 83 °C, slightly lower than the estimated (92 °C) based on the classical formula for long DNA sequences (Von Ahsen et al. 2001) and GenBank accession number M77789.2 for pUC19 sequence.

**Table 1:** Calculated thermal denaturation parameters from SRCD

Biomolecule	T_m_ (°C)	ΔH_m_ (kcal/mol) (×10^− 2^)
pUC19	83 ± 1	1.0 ± 0.1
DgrDps ΔN	70 ± 1	1.4 ± 0.2
DgrDps WT	67 ± 1	0.9 ± 0.1

The DgrDps proteins, WT and ΔN, exhibit similar thermal denaturation profiles, with T_m_ values of approximately 67 °C and 70 °C, respectively. The slightly lower T_m_ for the WT protein may be attributed to its long, flexible and solvent-exposed N-terminal tails (12 in total). These may facilitate the denaturation process by reducing the overall stability of the nanocage structure, thus requiring less energy to destabilize the native structure of the protein, as further supported by the calculated ΔH_m_ values. The T_m_ of DgrDps is comparable to the value of 69.2 ± 0.1 °C reported for DrDps1 (Grove and Wilkinson 2005).

### Characterization of DgrDps-pUC19 complexes

**Fig. 9:**
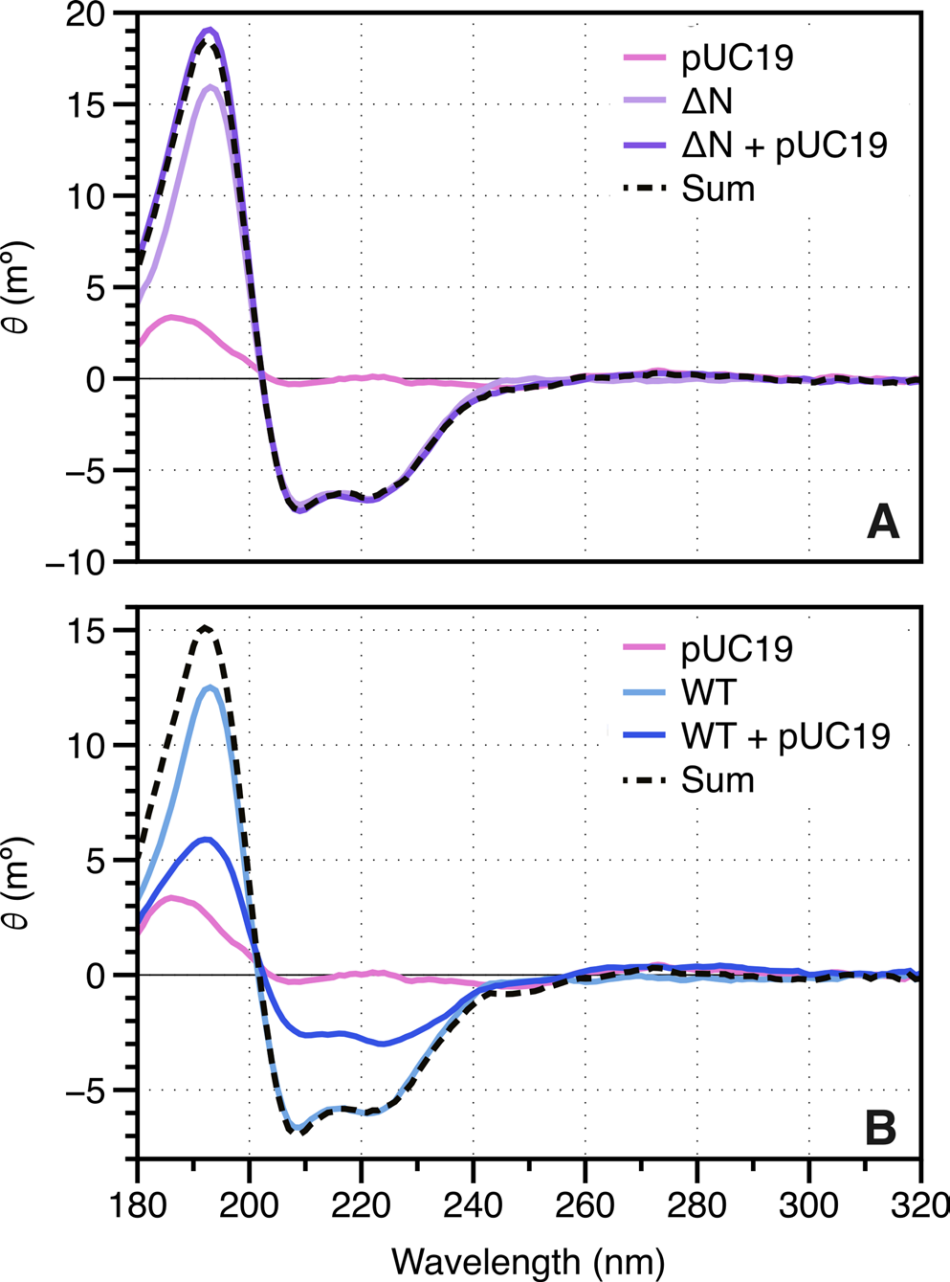
– SRCD characterization of DgrDps proteins (WT and ∆N) and pUC19 complexes. (A) Spectra of DgrDps ΔN (0.3 mg/mL), pUC19 (0.1 mg/mL), the binding reaction mixture, and the algebraic sum of the individual DgrDps ΔN and pUC19 spectra. (B) Spectra of DgrDps WT (0.3 mg/mL), pUC19 (0.1 mg/mL), the binding reaction mixture, and the algebraic sum of the individual DgrDps WT and pUC19 spectra

To further elucidate the interaction between DgrDps proteins (WT and ∆N) and pUC19, we initially compared the SRCD spectra of the protein-DNA binding reaction mixtures, recorded at 24 °C, against the spectra of the isolated protein and DNA components (Fig. 9). When pUC19 was incubated with DgrDps ΔN, the resulting SRCD spectrum coincided with the composite spectrum obtained by the sum of the individual protein and DNA spectra (Fig. 9A). This result indicates that mixing DgrDps ∆N with the plasmid DNA led to no detectable structural changes, as measured by SRCD. In contrast, the reaction of DgrDps WT with pUC19 significantly decreased the overall intensity of the CD spectrum, a change not explainable by a simple additive effect of the spectra of the individual components (Fig. 9B). Absorbance spectra of the two proteins, pUC19 and their corresponding mixtures in the significant range of 235 to 320 nm (Figure S1) indicate no loss of b content due to aggregation or similar effects. A similar effect was previously reported when using MnDps (4 µM) and pUC19 (125 nM) (Jacinto et al. 2021). However, the signal decrease for the DgrDps-pUC19 mixture was considerably more pronounced, suggesting a greater change in the molecular structure of the complex.

**Fig. 10:**
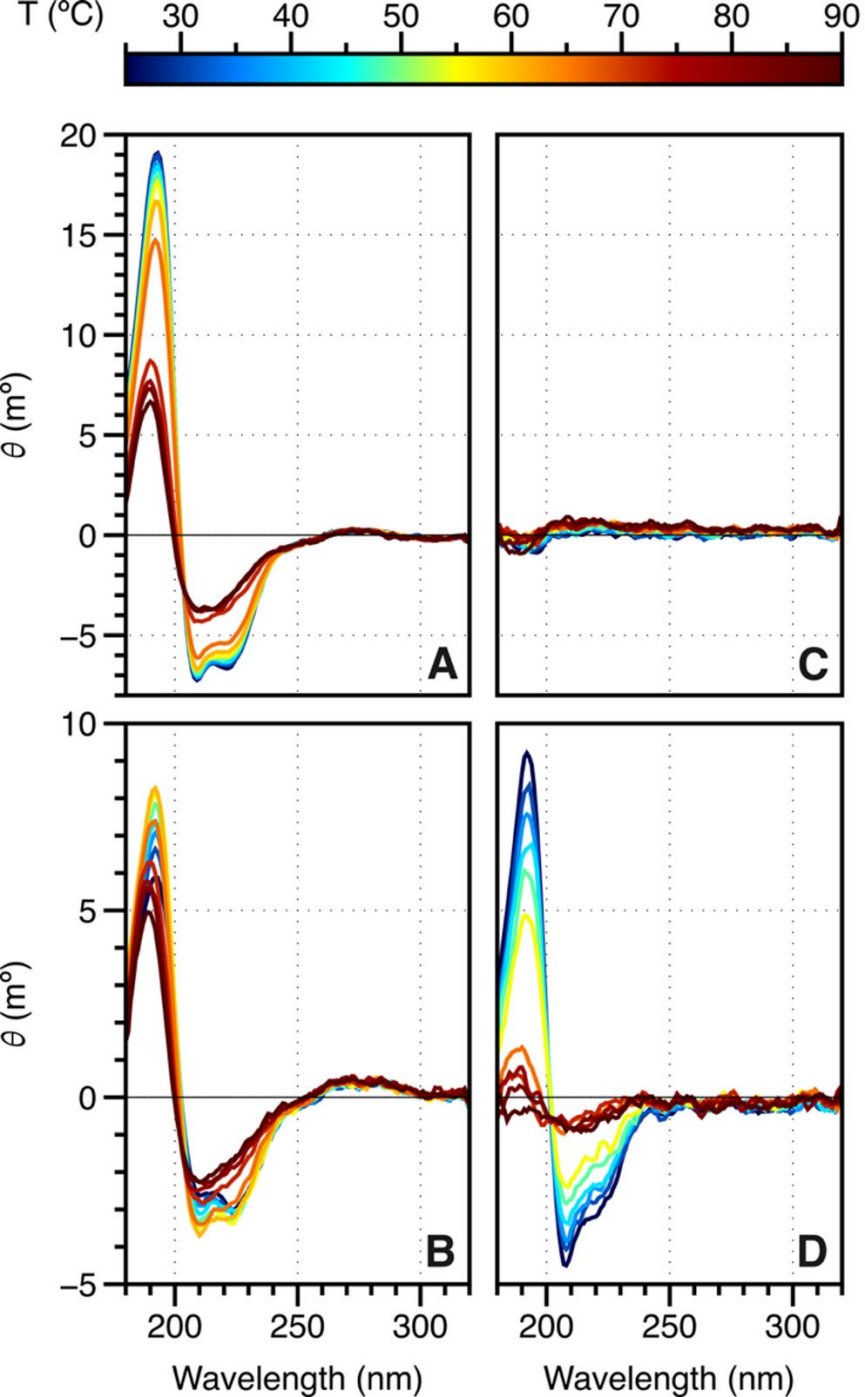
– Investigating DgrDps proteins and pUC19 interactions via SRCD thermostability analysis. Left panels: Experimental SRCD spectra from the binding reaction samples of DgrDps ΔN variant (A) and WT (B) with pUC19. Right panels: Difference spectra for DgrDps ∆N (C) or WT (D), from those measured with pUC19. Spectral changes observed across the temperature scan obtained by subtracting the sum of the independent components from their corresponding experimental spectra presented in panels A and B, respectively. Samples contained DgrDps proteins, WT and ∆N, at 0.3 mg/mL and pUC19 at 0.1 mg/mL in 10 mM MOPS, pH 7.0 and 240 mM NaF

Given the limited existing information in the literature on these protein-DNA interactions, we opted to conduct a deeper study using SRCD. Temperature scans (24 to 87 °C) were performed on DgrDps WT and ∆N proteins, both individually and when mixed with pUC19. The spectra from these samples were then compared to the composite spectra obtained by linear combination of the individual protein and pUC19 components recorded at corresponding temperatures (Fig. 10). A negligible difference was observed between the sum spectra of the individual pUC19 and DgrDps ΔN components and the experimental protein-DNA mixture, confirming an additive spectral behavior (Fig. 10A and C). This corroborates the lack of interaction at all temperatures, indicating that both protein and DNA molecules behave independently, as expected from the individual temperature-dependent experiments performed. For DgrDps WT, notable differences emerged between the mathematical composite of the components’ spectra and that of the experimental mixture. These dissimilarities are presumed to reflect the concentration of protein-DNA complex formed in solution across the temperature range.

Careful analysis of the SRCD spectrum at 24 °C shows two key features: (i) ellipticity in the 180–200 nm range is less intense, and (ii) the ellipticity ratio at 208/223 nm is markedly different (> 1) compared with either the protein alone or the protein–DNA mixture with the ∆N variant.

**Fig. 11:**
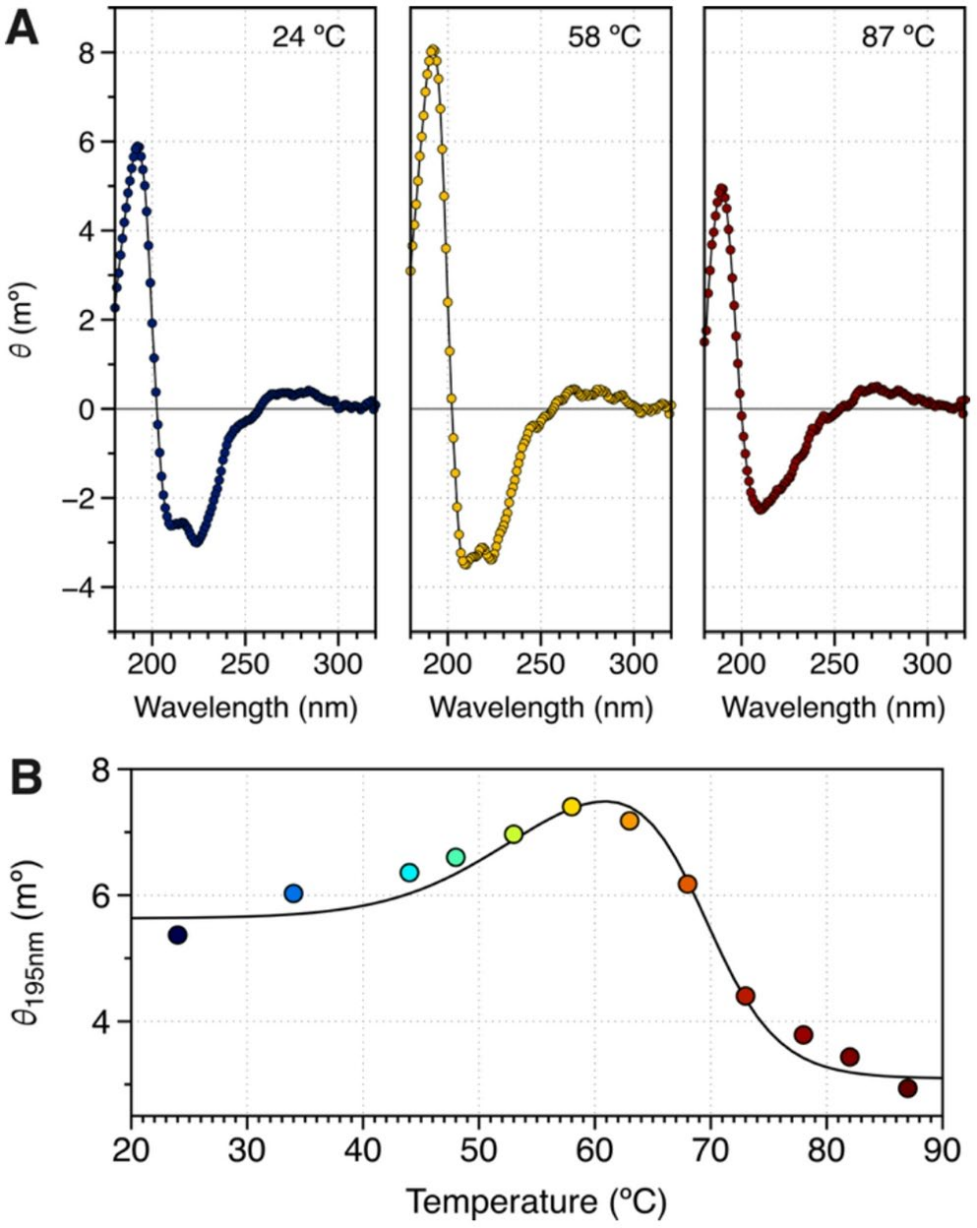
– Temperature evolution of the DgrDps WT-pUC19 mixture monitored by SRCD. (A) Experimental SRCD data at 24 °C, 58 °C, and 87 °C. (B) Circles represent the ellipticity at 195 nm over temperature. The solid line represents a weighted simulation calculated using the estimated parameters (T_m_ and ΔH_m_) obtained from Figure S2 for the two transitions observed. This two-step process is composed by three species: protein bound to DNA, free native protein and denatured protein

Upon increasing the temperature from 24 to 60 °C, this spectral feature is progressively lost, with the 208/223 nm ratio shifting towards the values observed for the protein alone (< 1). Ellipticity at 195 nm increases in intensity up to 60 °C (Fig. 11). Above this temperature, the negative double peak at 208 and 223 nm disappears, giving rise to a single, weaker peak at 210 nm, accompanied by a concomitant decrease in ellipticity at 195 nm (Fig. 11). These spectral changes reflect two successive processes: an initial complex dissociation favored between 24 and 60 °C, followed by protein denaturation above 60 °C.

To gain more insight into the thermal stability of the protein-DNA complex and profiting from the particular 208 and 223 nm spectral features, we performed weighted difference ellipticity calculations as a function of temperature thus obtaining temperature profiles for both effects (see Figure S2). From these profiles, we obtained ΔH_m_ and T_m_ of 37 ± 3 kcal/mol and 54 ± 1 °C, corresponding to the thermal stability of the protein-DNA complex. The ΔH_m_ value is at least halved relative to that obtained for the DgrDps WT and variant proteins. This likely reflects the combined effects of disrupted hydrogen bonding and other non-covalent interactions, together with the more favorable hydration of both plasmid and protein molecules compared with the protein-DNA complexes in solution. Such an effect was already seen in Dps protein fibers (Pacheco et al. 2020). The additional information obtained showed ΔH_m_ and T_m_ values of 75 ± 4 kcal/mol and 69 ± 1 °C. These values, within the error of measurement, are equal to the those obtained for the DgrDps WT thermal denaturation process, supporting the interpretation of the data. Furthermore, using the complete parameter set and a simple three-state model (Almeida et al. 2021), we were able to predict the ellipticity progression at 195 nm (Fig. 11, panel B), which accounts for both effects described.

## Conclusions

Dps proteins were initially discovered due to their ability to bind DNA under conditions of bacterial nutritional stress. However, it is now clear that not all members of this protein family share this DNA-binding capability. The non-specific DNA-binding ability of Dps has commonly been attributed to their positively charged N- or C-terminal tails, which interact with the phosphate groups of the DNA backbone. Despite this general understanding, the specific modes of Dps-DNA binding can vary depending on the protein and microbial origin.

In this study, we evaluated the DNA-binding capabilities of *Deinococcus grandis* Dps (DgrDps). We examined both the WT form and a variant lacking the N-terminal tails (∆N), using the pUC19 plasmid as a model DNA target. EMSAs showed that DgrDps WT binds to supercoiled plasmid DNA with a *K*_D_ of 5.2 ± 0.3 µM, also exhibiting positive cooperativity. The flexible N-terminal tails proved to be essential for DNA interaction, as the ΔN variant was unable to bind pUC19. This result aligns with previous observations for other Dps homologues (Bhattacharyya and Grove 2007). The effect of iron loading on DNA binding was also assessed for DgrDps WT. Under the experimental conditions, although the 96 Fe^2+^-loaded protein revealed a dissociation constant similar to that of the apo-protein, the presence of an iron mineral core significantly favored protein-DNA condensation. This observed effect is consistent with previous findings for other Dps proteins, highlighting their dual mode of DNA protection: they act as ferritin-like proteins by removing the toxic Fe^2+^ ions from the medium, and physically shield the DNA molecule.

The formation of protein-DNA complexes was crucial for DNA protection against DNase I degradation, as our results demonstrated that Dps-DNA complexes physically shield the bound DNA from nuclease activity. However, protease assays using Proteinase K revealed no protection for the protein itself. These results were additionally corroborated by AFM imaging, which revealed the formation of large protein-DNA aggregates and significant DNA condensation through protein aggregation. This mode of interaction exposes Dps to the solvent while protecting the genetic material.

SRCD spectroscopy was initially employed to probe the structural integrity and thermal stability of both WT and ∆N mutant DgrDps proteins, as well as pUC19. The collected data confirmed that both proteins retained the expected secondary structure characteristic of the Dps nanocage. Additionally, the spectral features of pUC19 were consistent with those described in the literature. The SRCD spectra obtained from incubating each of the DgrDps proteins with pUC19 revealed distinct behaviors for the two protein variants. The DgrDps ΔN–pUC19 reaction mixture displayed spectral superposition, with the composite spectra closely matching the algebraic sum of the individual components (ΔN and pUC19) across all tested temperatures, indicating no interaction in solution, while WT-pUC19 incubation yielded strikingly different results. The most noticeable difference was a decrease in overall CD signal intensity, suggesting a structural perturbation upon protein-DNA complex formation. In addition, spectral changes occur in the 200 to 225 nm wavelength region, displaying inverse intensity for the negative peaks in that region. Such spectral changes could be due to several effects attributed to protein-DNA complex formation. For example, protein binding may partially disrupt α-helices (e.g. helix-to-coil transitions upon binding) thus reducing ellipticity. DNA binding with additional hydrogen bonding to DNA bases or polar DNA backbone could cause electronic coupling with peptide bond transitions and alter local dielectric environment which ultimately may also be responsible for the lower/changed ellipticity observed. Importantly, the characteristic changes in ellipticity provide a means to specifically follow the interaction between the protein and DNA. It was, thus, possible to characterize the thermal stability of the complex with a calculated T_m_ value of 54 ± 1 °C. Using this value we can predict a threshold of 39 to 43 °C for the initial complex destabilization (5 to 10%). Interestingly, it was recently described that upper permissive temperature limit for *D. grandis* growth is 40 °C (Sakai et al. 2024), which gives additional relevance for the study of these molecular interactions and the study of DNA protection by Dps proteins, in particular for thermophile microorganims.

In sum, the present work not only allowed the characterization of the protein-DNA mixture but also provided an analytical tool for investigating this interaction in greater detail. Despite significant advances in understanding Dps-DNA interactions, much remains to be explored, particularly concerning different DNA topologies and sizes. As the precise mechanism underlying Dps binding to various DNA types is still not fully understood, further studies to clarify this important process are required.
